# Learning curve of blood pressure management during phaeochromocytoma surgery without preoperative alpha-blockade dose escalation

**DOI:** 10.1530/EC-26-0158

**Published:** 2026-08-25

**Authors:** Isabelle Holscher, Mahsoem Ali, Koen M A Dreijerink, Markus W Hollmann, Tijs J van den Berg, Wouter D Lubbers, Els J M Nieveen van Dijkum, Anton F Engelsman

**Affiliations:** ^1^ Amsterdam UMC Location University of Amsterdam, Department of Surgery, Amsterdam, The Netherlands; ^2^ Amsterdam Gastroenterology Endocrinology Metabolism, University of Amsterdam, Amsterdam, The Netherlands; ^3^ Amsterdam UMC Location Vrije Universiteit Amsterdam, Department of Endocrinology and Metabolism, Amsterdam, The Netherlands; ^4^ Amsterdam UMC Location University of Amsterdam, Department of Anesthesiology, Amsterdam, The Netherlands

**Keywords:** adrenalectomy, CUSUM analysis, α-blockade non-escalation, perioperative haemodynamic

## Abstract

**Background:**

Adrenalectomy without preoperative dose escalation of α-blockade is gaining popularity for managing phaeochromocytoma, showing comparable outcomes but shorter hospital stays and less postoperative hypotension. However, recent data showed an initial significant increase in intraoperative hypotension after transitioning to this approach, suggesting a learning curve. We evaluated this learning curve for the safe use of our α-blockade non-escalation workflow employing a risk-adjusted CUSUM (RA-CUSUM) analysis.

**Method:**

This single-centre cohort study included phaeochromocytoma resections without preoperative α-blockade dose escalation from 2019 until 2023. All procedures were performed by an experienced operating theatre team, including anaesthesiologists and surgeons dedicated to phaeochromocytoma treatment. Intraoperative hypotension (time-weighted average (TWA) of mean arterial pressure (MAP) < 60 mmHg) was identified as primary learning curve outcome. RA-CUSUM analysis consisted of predicting intraoperative hypotension using a multivariable linear regression model corrected for established risk factors and modelling the relationship between the number of procedures and the cumulative difference in predicted and observed outcomes.

**Results:**

A total of 44 procedures were evaluated employing the new preoperative α-blockade non-escalation strategy. RA-CUSUM analysis showed that the learning curve was completed after 19 surgeries. The median TWA of MAP < 60 mmHg was 2.29 (IQR: 0.45–4.16) mmHg. No perioperative complications related to haemodynamic instability occurred in the cohort. Time in the operating theatre and length of hospital stay remained consistent over time.

**Conclusion:**

Our results confirm that transitioning to phaeochromocytoma resection without α-blockade dose escalation follows a learning curve. To ensure safe implementation, centres adopting the non-escalation workflow should meet certain conditions, with structured guidance or proctoring as possible measures.

## Introduction

Adrenalectomy without preoperative dose escalation of α-blockade is gaining popularity in the surgical management of phaeochromocytoma (1, 2). Traditionally, preoperative α-blockade dose escalation was routinely used to reduce the risk of haemodynamic instability and associated cardiovascular complications caused by catecholamine release during surgery (3, 4, 5, 6, 7, 8). Despite the widespread use and guideline endorsement of α-blockade dose escalation as standard preoperative care, increasing evidence emphasises its potential drawbacks, including prolonged postoperative hypotension due to persistent vasodilatory effects (1, 2, 9, 10, 11, 12, 13, 14, 15). This realisation has resulted in a shift towards adrenalectomy without routine preoperative dose escalation of α-blockade. Early data suggest that this non-escalated management is a safe and effective alternative when accompanied by adequate intraoperative monitoring and administration of short-acting vasoactive agents, showing comparable perioperative complication rates, but less postoperative hypotension and shorter hospital stays (16, 17, 18).

Despite the promising findings regarding the non-escalation strategy, the implementation of this approach is not simply a matter of omitting preoperative α-blockade dose escalation. The transition involves a shift in intraoperative haemodynamic management, which requires an experienced and dedicated operating room team, including anaesthesiologists and surgeons, to adjust accordingly. However, limited data have been reported on this process of implementation and the possible learning curve associated with transitioning to this non-escalation workflow. In our single-centre study, we observed an initial increase in intraoperative hypotension, suggesting that adoption of the non-escalation strategy requires a learning period regarding intraoperative blood pressure management (17). This observation was based on a comparison of hypotension rates between two equally divided subgroups within the preoperative non-escalated protocol group: an early (inexperienced) and a later (experienced) group. However, this comparison did not account for confounding factors that may have influenced outcomes independently of team experience and lacked a statistical evaluation of performance over time. Accordingly, it cannot be considered formal evidence of a learning curve.

To accurately assess whether a learning effect was present, a structured learning curve analysis is necessary. Identifying this learning curve is essential for optimising the implementation of the new strategy and ensuring a safe transition for other operating theatre teams. The aim of this study was to assess the potential learning curve associated with the implementation of adrenalectomy without preoperative dose escalation of α-blockade, using a risk-adjusted cumulative sum (RA-CUSUM) analysis to evaluate changes in performance over time while correcting for potential differences in risk factors. Additionally, it should be noted that the specific perioperative challenges of this surgical approach may not be adequately captured by commonly used indicators such as operative time (19, 20, 21).

We therefore assessed multiple intraoperative outcome measures to determine which parameter most appropriately reflects the learning process. We hypothesise that the transition to adrenalectomy without routine α-blockade dose escalation is associated with an initial learning curve and that intraoperative haemodynamic parameters may better reflect this learning process than operating time.

## Materials and methods

### Study design and data source

This study used data from the prospectively maintained database of a single-centre study that included patients undergoing phaeochromocytoma resection between December 2015 and August 2023. This database cohort was previously studied by our team to evaluate clinical outcomes after changing the preoperative strategy from α-blockade dose escalation to a non-escalation approach, by comparing perioperative outcomes between a historical dose-escalated group and a prospectively monitored non-escalated group (17). From this database, all consecutive patients receiving the revised non-escalation preoperative protocol without dose escalation were included. The non-escalation preoperative protocol, implemented in 2019, replaced the historical dose escalation strategy for all phaeochromocytoma surgeries with only initiation of low-dose doxazosin (4–8 mg) at diagnosis when antihypertensive treatment was indicated. Patients were admitted less than 24 h before surgery. Intra- and postoperative management remained unchanged between protocols. The surgical approach was not standardised. Throughout the study period, no modifications were made to the non-escalation protocol or perioperative management.

All procedures were performed by our phaeochromocytoma surgery expert team, consisting of two endocrine surgeons and two anaesthesiologists with substantial experience in phaeochromocytoma management prior to implementation of the non-escalation strategy. In the current study, all operating room team compositions were included, comprising our expert team as well as other experienced anaesthesiologists in fewer than 10% of cases. Phaeochromocytoma surgery is a multidisciplinary effort in which outcomes, such as intraoperative haemodynamic instability, depend not only on individual expertise, but also on team communication and coordination. To reflect this team-based dynamics and minimise selection bias, we evaluated the learning curve across the entire surgical cohort, instead of isolating the performance of a single anaesthesiologist or surgeon.

Patient inclusion required a postoperative pathology-confirmed phaeochromocytoma diagnosis. Exclusion criteria included missing data on preoperative treatment or incomplete pre-treatment, unrelated severe comorbidities (e.g. congestive heart failure, concurrent cancer), metastatic disease and complex surgeries beyond the standard scope of phaeochromocytoma cases (e.g. arterial or organ reconstructions due to severe tumour adhesions). This study was conducted under ethical review by the local Medical Ethics Review Committee and adheres to the Strengthening the Reporting of Observational Studies in Epidemiology (STROBE) guidelines (22).

### Primary outcome measure

Correlation and regression analyses were performed to identify the most appropriate outcome measure for assessing the learning curve under the non-escalation workflow. For these analyses, both haemodynamic and clinical parameters were evaluated, including time-weighted averages (TWAs) of systolic blood pressure (SBP) and heart rate deviations beyond clinically relevant thresholds (SBP > 200 mmHg, mean arterial pressure (MAP) < 60 mmHg, heart rate > 100 bpm or < 60 bpm), postoperative norepinephrine duration, intraoperative blood loss and operative time parameters (surgery time, overall operating room duration and time from intubation to incision). The haemodynamic thresholds were selected based on previous literature identifying these values as the most commonly used and clinically relevant definitions in phaeochromocytoma surgery (23). All haemodynamic outcome measures were expressed as TWAs to account for both the duration and intensity of haemodynamic instability, providing a more comprehensive measure than peak values or duration alone. TWA was calculated by summing the deviations above or below the predefined thresholds (e.g. the sum of deviation magnitudes in mmHg above 200 mmHg) and dividing this value by the total surgical duration. Intraoperative hypotension (TWA of MAP < 60 mmHg) showed the strongest correlation with perioperative team experience in the non-escalation cohort and was selected as the primary outcome. Potential predictors of intraoperative hypotension were assessed using univariable analysis (*P* < 0.20), followed by multivariable linear regression (*P* < 0.05) with manual backward selection (see Supplementary Table S1 (see section on Supplementary materials given at the end of the article) for a list of tested parameters). Final variable selection was based on statistical significance, clinical relevance and a comparison of adjusted R-squared values between models to ensure optimal fit. Categorical variables were excluded from the analysis if there were fewer than 10 patients in a specific category.

### Statistical analysis

Baseline characteristics are presented as mean (SD) in the case of normally distributed continuous data and median (IQR) in the case of non-normally distributed continuous data. Categorical variables are presented as absolute numbers (%). The overall learning curve of the operating theatre team was investigated employing the CUSUM method, a statistical tool used to evaluate performance over time by plotting the difference between the cumulative expected outcome and the actual outcome. In the risk-adjusted CUSUM (RA-CUSUM), the expected outcome was predicted by a multivariable linear regression model. This analysis was conducted in three stages: i) predicting the primary outcome (TWA of MAP < 60 mmHg) based on prespecified predictors, ii) calculating the difference between the predicted and observed outcome and iii) modelling the relationship between the number of procedures and the cumulative difference in predicted and observed outcomes. First, multivariable linear regression analysis was used to predict the expected TWA of MAP < 60 mmHg, based on the predefined predictors that were significantly different in our multivariable analysis. Modelling with restricted cubic splines was used in the case of a non-linear relationship with the outcome. To assess whether this model systematically over- or underestimated the outcome, bootstrap resampling based on 1,000 bootstrap samples was used to create overfitting-corrected calibration curves. These calibration curves show the agreement between the predicted and observed TWA of MAP < 60 mmHg. Ideally, this curve has an intercept of 0 and a slope of 1, indicating perfect agreement between the predicted and observed outcomes.

For the risk-adjusted CUSUM analysis, the cumulative difference between the expected outcome based on the regression model and the observed outcome was calculated and plotted in chronological order of the surgical procedures. An upward slope indicates performance worse than expected, while a downward slope reflects improvement compared with the risk-adjusted model. A smooth trend line for the relation between the number of procedures and the cumulative difference in expected and observed outcome was estimated using linear regression analysis, in which the number of procedures was non-linearly modelled with restricted cubic splines. This trend line was used to determine the inflection point, indicating the point at which the learning curve had been completed.

### Software

Statistical analyses were performed using Statistical Package for the Social Sciences (SPSS, version 28.0, IBM Corp., USA) and R (R Foundation for Statistical Computing). A *P* value less than 0.05 was considered to be statistically significant.

## Results

### Baseline characteristics

A total of 44 procedures were conducted employing the new management without preoperative dose escalation (see Supplementary Fig. S1 for the study flowchart). As mentioned in our previous study, baseline demographic and tumour characteristics of this non-escalation cohort were similar to our historical cohort of patients receiving preoperative dose escalation of α-blockade, indicating that the non-escalation cohort of patients is representative of the general phaeochromocytoma population. Baseline characteristics are described in Table 1.

**Table 1:** Baseline characteristics.

Patient and tumour characteristics	Non-escalated protocol cohort (*n* = 44)
Sex (F/M)	24/20
Age (years)	56.6 (13.3)
BMI (kg/m^2^)	25.6 (23.7–28.3)
Symptomatic patients (*n*)	33 (75.0%)
Diabetes (*n*)	10 (22.7%)
Prior cardiovascular event (*n*)	14 (31.8%)
Germline mutation (*n*)	
Yes*	7 (15.9%)
No	8 (18.2%)
No genetic testing performed	29 (65.9%)
Biochemical levels (nmol/L)	
Dominant secretion	
Metanephrine	14 (31.1%)
Normetanephrine	30 (66.7%)
Plasma metanephrine^†^	1.05 (0.26–5.24)
Plasma normetanephrine^‡^	4.77 (1.69–11.21)
Plasma 3-MT^§^	0.09 (0.00–0.18)
Tumour localisation	
Left	22 (50.0%)
Right	22 (50.0%)
Tumour size (mm)^║^	48.0 (30.0–67.8)
Surgical procedure	
Open (*n*)	2 (4.5%)
Minimally invasive surgery (*n*)	42 (95.5%)
Transabdominal	41
Retroperitoneal posterior	1
Surgical conversion	2 (4.5%)

Values are presented as median (IQR), mean ± SD, or *n*/number of patients (%), as appropriate.

* MEN1 in 1 patient, MEN2A in 1 patient, NF1 in 2 patients, VHL in 2 patients and SDHB mutation in 1 patient.

^†^ Plasma metanephrine levels: 4 missing values. Reference range: 0–0.45 nmol/L.

^‡^ Plasma normetanephrine levels: 4 missing values. Reference range: 0–1.05 nmol/L.

^§^ Plasma 3-MT levels: 9 missing values. Reference range: 0–0.10 nmol/L.

^║^ Tumour size: 1 missing value.

### Perioperative outcomes

**Figure 1:**
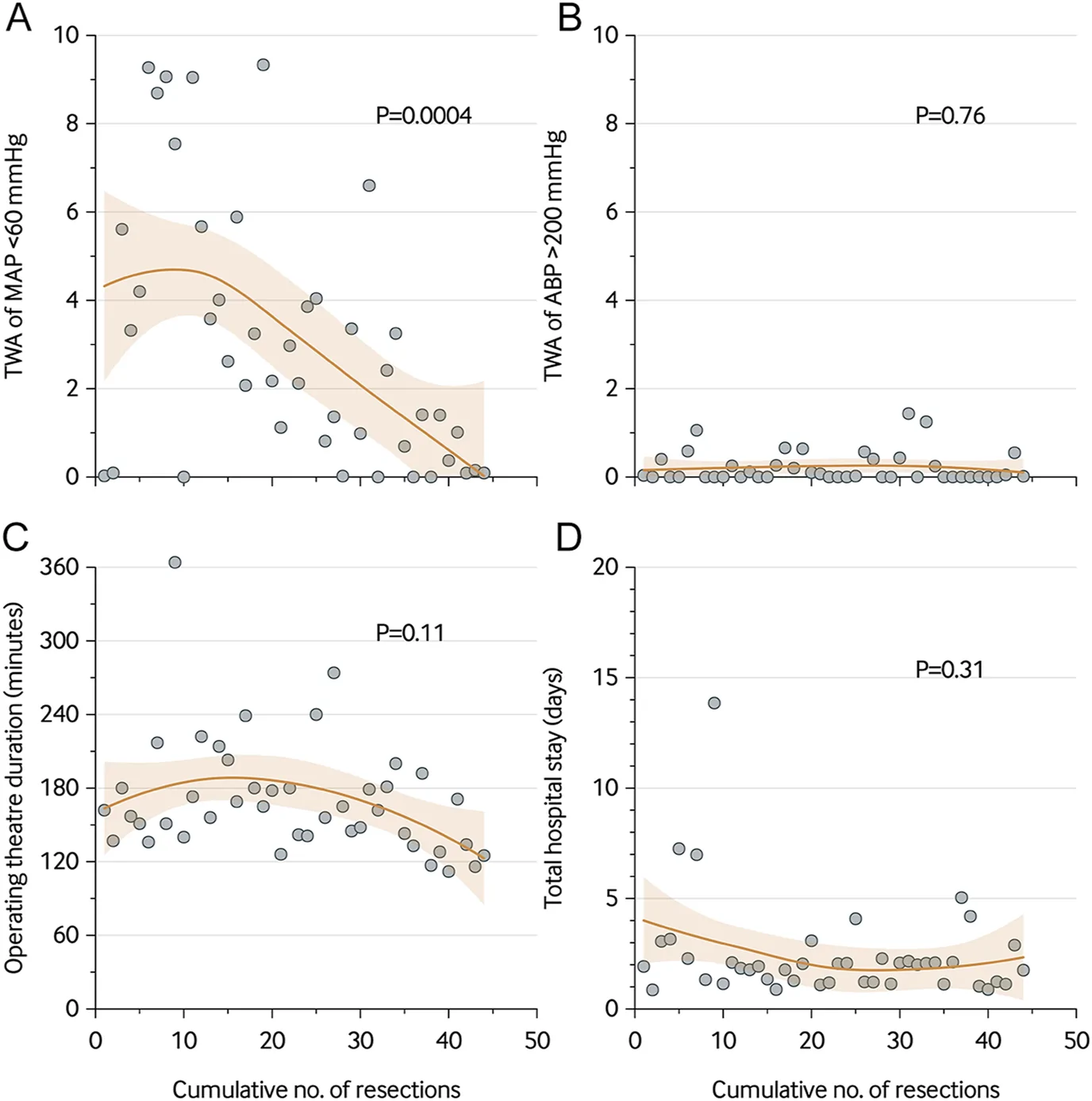
Correlation of surgical outcomes with operating theatre team experience.

Of the 44 procedures, 42 (95%) were initiated laparoscopically, with one case requiring conversion to open surgery. The median time in the operating theatre was 162 (IQR: 140–181) min, with a surgical time of 80.5 (IQR: 63–101) min. The median total length of hospital stay was 2.5 days (1.9–3.6), with a median preoperative admission duration of 0.5 days (IQR: 0.2–0.8) and postoperative duration of 2.0 days (1.2–2.3). Data on occurrence and duration of intraoperative hypotension are described in Supplementary Table S2. Figure 1 shows the correlation of operating theatre team experience with the variables intraoperative hypertension, defined as TWA of SBP > 200 mmHg, intraoperative hypotension (TWA of MAP > 60 mmHg), duration of surgery and length of hospital stay. Intraoperative hypertension, duration of surgery and length of hospital stay remained consistent over time and were therefore not correlated with operating theatre team experience. In the cohort, no perioperative complications related to haemodynamic instability were observed. Two patients had postoperative complications unrelated to haemodynamic instability. One patient experienced bleeding in a renal vein branch, and the patient who required intraoperative conversion to open adrenalectomy due to a preoperative haemorrhage in the operating area postoperatively developed kidney necrosis, necessitating resection of the affected kidney. No patients died within the first 90 days following surgery.

### Prediction model for intraoperative hypotension

In the multivariable model, both perioperative blood loss (*P* = 0.006) and duration in the operating theatre (*P* = 0.002) were significant predictors of intraoperative hypotension (Supplementary Table S1). A non-linear relationship between perioperative blood loss and intraoperative hypotension (*P*_non-linearity_ = 0.006) was found. The adjusted *R*^2^ of this model was 0.37, and bootstrap-corrected calibration curves indicated that the model did not systematically over- or underestimate the TWA of MAP < 60 mmHg (Supplementary Fig. S2).

### Learning curve

**Figure 2:**
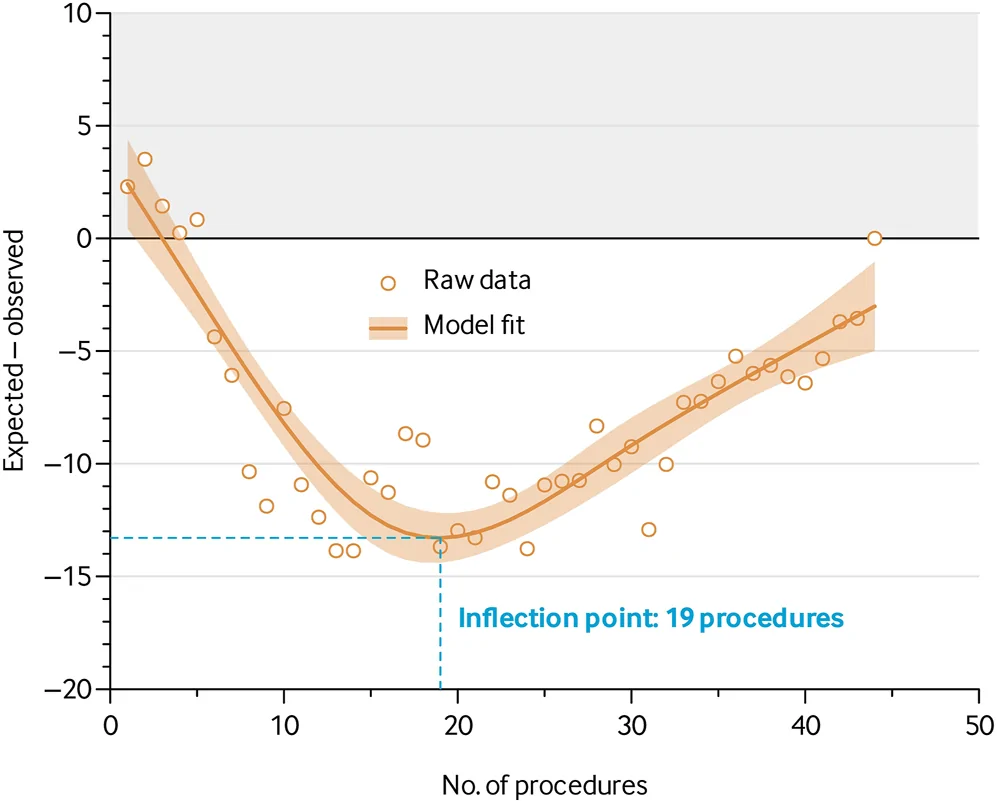
RA-CUSUM learning curve, showing the trend in performance across consecutive cases, adjusted for baseline risk factors.

The median overall TWA of MAP < 60 mmHg was 2.29 (IQR: 0.45–4.16) mmHg. Our learning curve plot shows that after 19 surgeries, the learning curve was completed (Fig. 2).

## Discussion

This single-centre study evaluated the learning curve associated with the transition to phaeochromocytoma surgery without α-blockade dose escalation. The risk-adjusted CUSUM analysis showed a learning curve of 19 procedures for this non-escalation strategy, adjusted for the risk factors time spent in the operating theatre and blood loss. Despite this learning phase, we found no increase in perioperative complications related to haemodynamic instability, indicating that adrenalectomy without preoperative dose escalation with α-blockade can safely be implemented.

However, our findings do highlight a significant learning curve regarding intraoperative blood pressure management, even in the presence of an operating theatre team with extensive expertise in phaeochromocytoma management. This finding emphasises the importance of acknowledging learning curves, even in contexts where they may not be immediately anticipated. Identifying and addressing such learning curves is critical for improving clinical practice and patient outcomes.

In our study, intraoperative hypotension (TWA of MAP < 60 mmHg) had the strongest correlation with surgical team experience. A plausible explanation for this initial increase in hypotension is that the surgical team initially focused strongly on preventing severe hypertension after discontinuing preoperative α-blockade dose escalation. For more than 50 years, preoperative α-blockade dose escalation has been considered essential to reduce the risk of intraoperative haemodynamic instability and associated cardiovascular complications (3, 4, 5, 6, 7). This long-standing routine may have led to an initial over-administration of vasoactive agents at the start of implementing the non-escalation approach, as shown by the higher dosages of administered vasodilating agents (Supplementary Table S3). These results illustrate the value of proctoring from centres experienced in phaeochromocytoma surgery without α-blockade dose escalation, allowing for a smooth transition to this non-escalation workflow. We furthermore anticipate that proctored implementation, second-degree learning and a dedicated and experienced anaesthesiology team will produce a learning curve significantly shorter than our first implementation learning curve of 19 procedures. We estimate that the learning curve for second- and third-generation learners could be reduced to fewer than 10 procedures.

Despite the initially observed increase in hypotension, no increase in perioperative complications was observed during the learning period. This finding may imply that diverging from stringent haemodynamic control does not necessarily increase perioperative complications and calls into question the identification of intraoperative haemodynamic instability as the primary predictive factor for perioperative morbidity. However, it should be noted that the revised protocol was introduced in our centre following knowledge exchange between our own anaesthesiologists and their colleagues in Essen, where procedures without preoperative α-blockade were already standard practice. This may have mitigated the risks associated with early implementation.

Regarding patient selection, there is currently no evidence supporting preoperative dose escalation, even for phaeochromocytoma patients at risk. At our centre, dose escalation was initially omitted only in patients with severe comorbidities or urgent need for surgery in acute cases, with successful outcomes. After these successful procedures, together with increasing evidence against preoperative dose escalation, the new non-escalation protocol was implemented as standard practice. Based on our observations, we do not believe that specific clinical characteristics should prevent the use of the non-escalation approach, although centres should remain aware of the associated learning curve during implementation.

It is important to acknowledge some limitations of this study. The single-centre study design may have limited the generalisability of our results, as the precise learning curve may vary across different institutions. Furthermore, although the size of the included cohort is of considerable significance given the rarity of phaeochromocytomas, the relatively small size in absolute terms reduces the precision of the estimated inflection point. Nevertheless, the mere presence of a learning curve itself, with a higher likelihood of haemodynamic instability at the beginning of the transition to non-escalation, is an important observation. This observation highlights the need for careful consideration when adopting the strategy. With adequate proctoring from experienced centres, however, a smooth transition and a shorter learning curve are likely achievable. The inclusion of intraoperative variables such as blood loss and operating theatre duration may be considered a limitation of the risk-adjusted CUSUM analysis, as they may not be fully independent of the primary endpoint. Although these variables were carefully considered to account for differences in procedural complexity independent of the team’s experience with the non-escalation protocol, this should be taken into account when interpreting the adjusted learning curve. A strength of this study is that it includes all operating theatre team compositions, rather than focusing on the learning curve of a single anaesthesiologist or surgeon. The clear learning curve observed in our study supports the idea that this is a collective operating theatre team effort and suggests that similar results could be expected in other centres. Another strength of our study was the thorough assessment of appropriate outcome measures when evaluating the learning curve, with adjustments for confounding predictors in the RA-CUSUM, allowing for correction of differences in case-mix over time.

In conclusion, our results confirm the presence of a learning curve during the transition to a preoperative strategy without dose escalation with α-blockade in phaeochromocytoma surgery, reflected by increased intraoperative hypotension during the early phase of implementation. Although this increase in hypotension did not result in perioperative complications in our cohort, hypotension is generally associated with adverse clinical outcomes (24, 25). To ensure safe implementation, centres adopting the non-escalation workflow may require meeting certain conditions, such as structured guidance or proctoring by an expert centre.

## Supplementary materials



## Declaration of interest

This manuscript has not been published and is not under consideration for publication elsewhere. Furthermore, this manuscript has been seen and approved by all authors. We have no conflicts of interest to disclose.

## Funding

This research did not receive any specific grant from any funding agency in the public, commercial or not-for-profit sector.

## Ethics

Data from this cohort study are available upon request from the corresponding author, Dr A F Engelsman. This study was approved by the local Medical Ethics Review Committee (METC Reference No. W19_428#19.494).
