# Supplementary material for: Learning curve of blood pressure management during phaeochromocytoma surgery without preoperative alpha-blockade dose escalation
Source: Endocr Connect. 2026 Aug 25;15(8):e260158. doi: 10.1530/EC-26-0158 (PMC13545747; doi:10.1530/EC-26-0158)

Supplementary Table 1 Results of univariable and multivariable linear regression analyses to identify potential confounders for inclusion in the RA-CUSUM analysis

Supplementary Table 1. Factors associated with TWA of MAP <60 mmHg

Factor	Univariable effect (95% CI)	P value	Multivariable effect (95% CI)	P value
**Sex (Female)**	1.01 (–0.78 to 2.80)	0.26		
**Age (yrs)**	–0.50 (–1.65 to 0.66)	0.39		
**BMI (kg/m^2)**	–0.17 (–1.17 to 0.84)	0.74		
**Preoperative plasma epinephrine (nmol/L)**	0.29 (–0.01 to 0.58)	0.06		
**Preoperative plasma norepinephrine (nmol/L)**	0.48 (–0.18 to 1.13)	0.15		
**Tumour size (mm)**	–0.03 (–1.13 to 1.07)	0.96		
**Tumour localization (left)**	–0.12 (–1.92 to 1.69)	0.90		
**OT duration (min)**	0.90 (0.18 to 1.62)	**0.016**	1.23 (0.48 to 1.98)	**0.002**
**Surgical blood loss (ml)***	Overall non-linear effect (χ^2=10.7)	**0.006**	Overall non-linear effect (χ^2=13.3)	**0.006**

Continuous variables were assessed for non-linearity using restricted cubic splines (RCS). For non-linear variables (*), χ^2 test p values are reported. The variables open surgery and conversion were considered for inclusion, but could not be included due to the low frequency (<2) of cases.
Abbreviations: OT, operating theatre.

Supplementary Table 2 Overview of intraoperative hypotension (mean arterial pressure <60 mmHg) for the different patient groups: the total non-escalation cohort and the non-escalation subgroups, divided in an 'inexperienced' group (before completion learning curve number of 19 procedures) and an 'experienced' group (after completion of the learning curve). Values are presented as median (IQR) or N/ Number of patients (%) as appropriate. The p-value represents the comparison between the two non-escalation subgroups.

**Intraoperative hypotension**	**Total non-escalation cohort (n=44)**		**Non-escalation 'inexperienced group' (n=19)**		**Non-escalation 'experienced group' (n=25)**		**P-value**
**Incidence**	40	(90.9%)	18	(94.7%)	22	(88.0%)	0.622
**TWA**	2.29	(0.45-4.16)	4.20	(2.62-8.69)	1.12	(0.12-2.69)	**<0.001**
**Duration**	48.5	(12.8-78.8)	81.0	(56.0-100.0)	26.0	(4.0-54.5)	**<0.001**
**Depth/min**	8.0	(4.3-11.6)	10.9	(8.0-13.9)	6.9	(3.3-10.0)	**0.006**
**Perc. OT time**	29.2	(10.41-42.0)	43.6	(28.5-54.6)	14.4	(3.0-30.7)	**0.001**

Abbreviations: MAP, mean arterial pressure; TWA: time-weighted average; OT, operating theatre.

Supplementary Table 3 Overview of intraoperatively administered vasoactive agents. Data are noted as median (IQR) or N / number of patients (%). P-value of median dosages are stated. In both groups, no patients received terlipressin or labetalol.

**Intraoperative vasoactive agents** (cases, %)	**Total non-escalated cohort (n=44)**		**non-escalated 'inexperienced' group (n=19)**		**non-escalated 'experienced' group (n=25)**		***P*-value**
	n (%)	*Median (IQR)*	*n (%)*	*Median (IQR)*	*n (%)*	*Median (IQR)*	
**Noradrenalin** (μg)	41 (93.2%)	610.1 (171.7-1196.7)	19 (100%)	868.4 (490.0-1928.0)	22 (88%)	467.3 (161.9-954.9)	0.077
**Adrenalin** (mg)	1 (2.3%)	0 (0-0)	0 (0%)	0 (0-0.03)	1 (4%)	0 (0-0)	0.315
**Phenylephrine** (μg)	24 (54.5%)	150 (0-600)	12 (63%)	400 (0-700)	12 (48%)	0 (0-550)	0.257
**Ephedrine** (mg)	2 (4.5%)	0 (0-0)	1 (5%)	0 (0-0)	1 (4%)	0 (0-0)	0.818
**Isoprenaline** (μg)	4 (9.1%)	0 (0-0)	4 (21%)	0 (0-0)	0 (0%)	0 (0-0)	0.068
**Nitroglycerin** (mg)	21 (47.7%)	0.5 (0-2.9)	11 (58%)	1.0 (0.0-3.5)	10 (40%)	0.3 (0.0-2.5)	0.323
**Nitroprusside** (mg)	18 (40.9%)	0.2 (0.0-4.3)	10 (53%)	1.0 (0.0-5.2)	8 (32%)	0 (0-0)	0.081
**Urapidil** (mg)	30 (68.2%)	10 (0.0-28.8)	15 (79%)	25 (10-75)	15 (60%)	10 (0-17.5)	0.004
**Phentolamine** (mg)	14 (31.8%)	0 (0-1.8)	3 (16%)	0 (0-0)	11 (44%)	0 (0-0)	0.099
**MgSO4** (g)	40 (90.9%)	5 (4-5)	17 (90%)	5 (4-7)	23 (92%)	5 (3.8-5)	0.076
**Nicardipine** (μg)	1 (2.3%)	0 (0-0)	0 (0%)	0 (0-0)	1 (4%)	0 (0-0)	0.212
**Metoprolol** (mg)	11 (25.0%)	0 (0-0.8)	8 (42%)	0 (0-3)	3 (12%)	0 (0-0)	0.019
**Esmolol** (mg)	31 (70.5%)	45.8 (0-269.0)	14 (74%)	146.8 (0-326.7)	17 (68%)	40.0 (0.0-253.2)	0.456

Supplementary Figure 1 Flowchart of patient inclusion

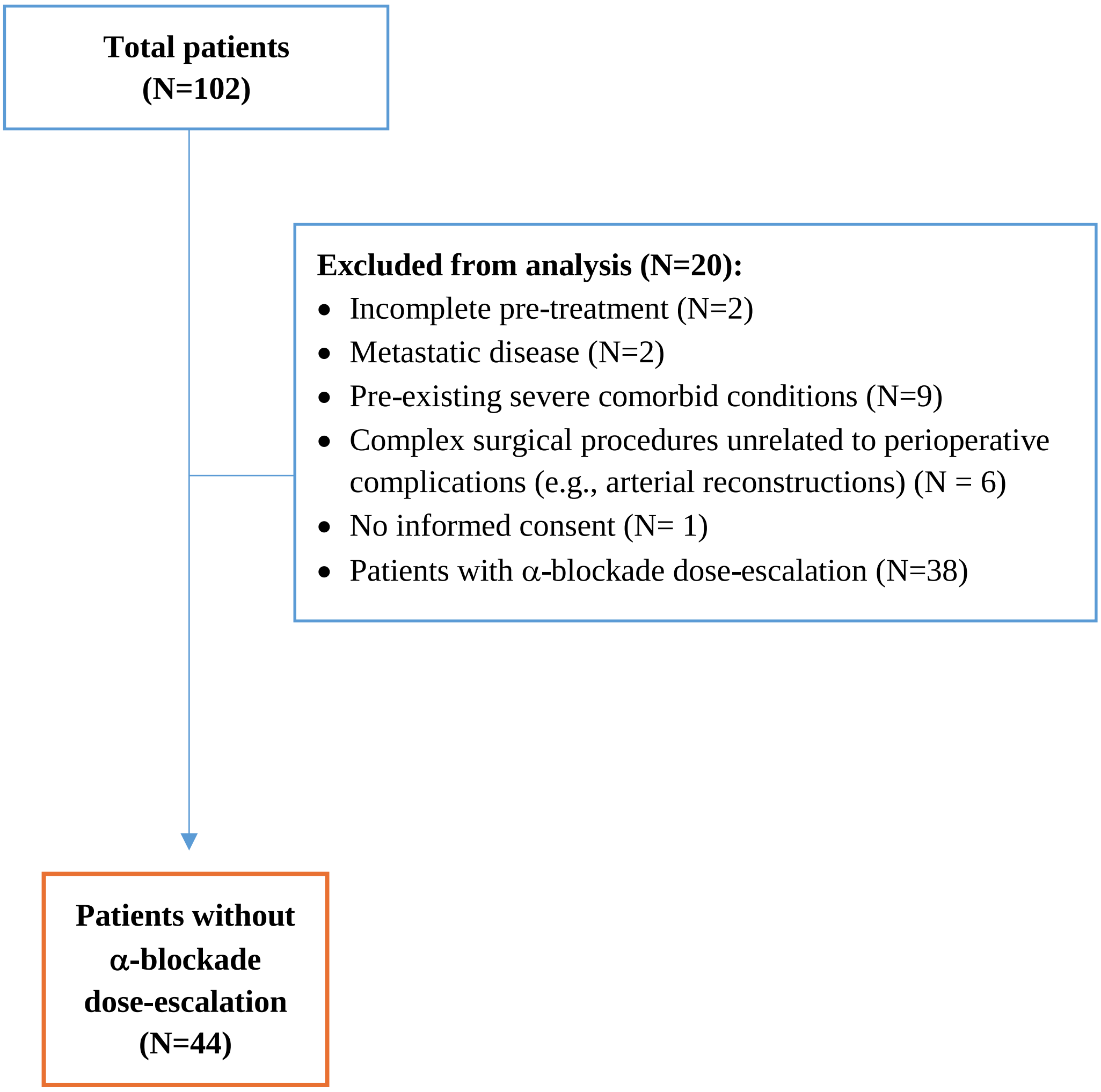

Supplementary Figure 2 Calibration Curve, showing the agreement between the predicted and observed TWA of MAP <60 mmHg of the model.

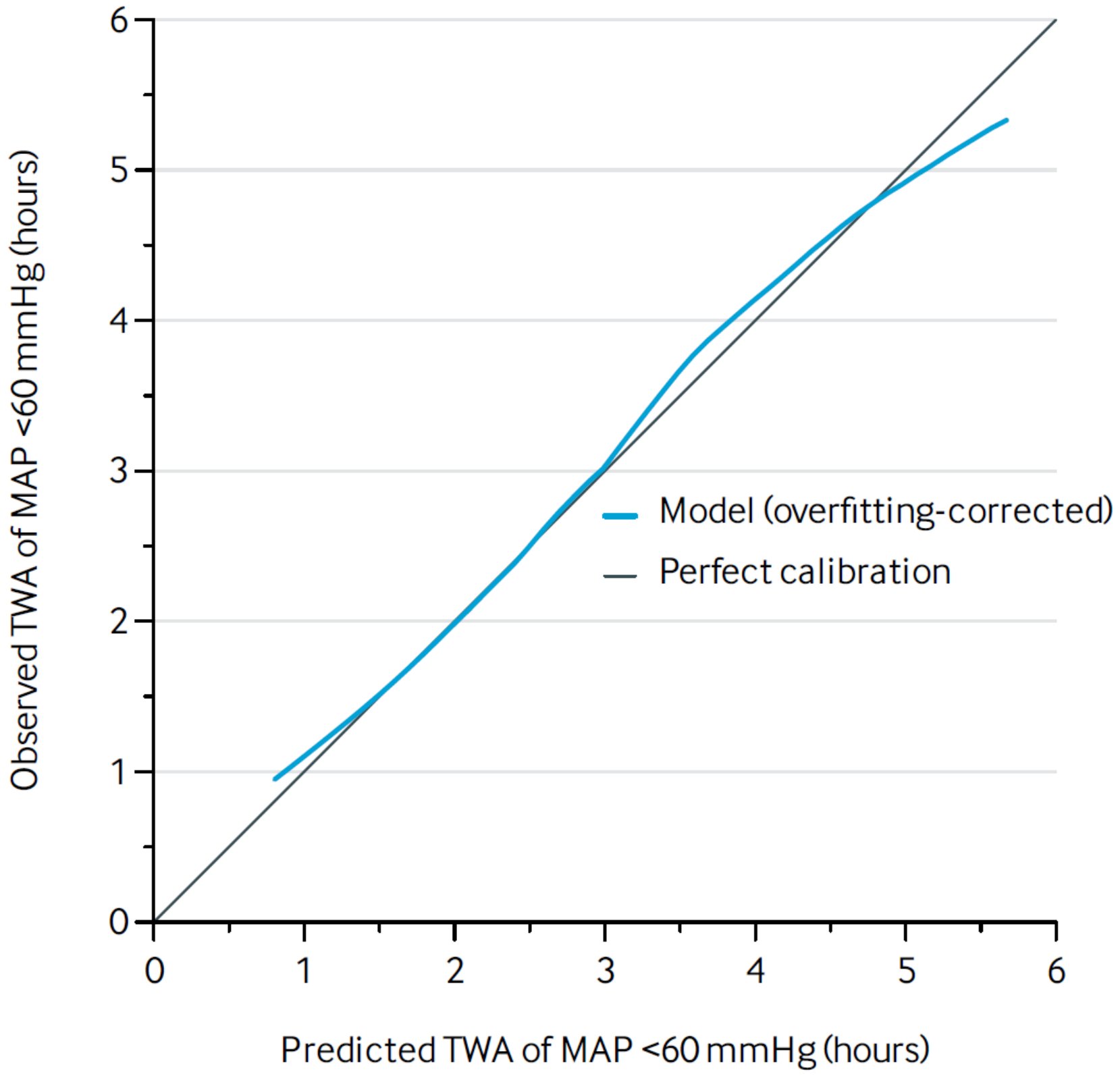

Supplement: Supplementary file 1 [file EC-26-0158_supplementary_materials.pdf]
